# Binostril endoscopic transsphenoidal neurosurgery for pituitary adenomas: experience with 42 patients

**DOI:** 10.18632/oncotarget.16976

**Published:** 2017-04-09

**Authors:** Guan Sun, Ying Cao, Nan Jiang, Dekang Nie, Zhengqiang Wan, Min Li, Chiyuan Ma, Jun Guo

**Affiliations:** ^1^ Department of Neurosurgery, The First People’s Hospital of Yancheng, Yancheng, P R China; ^2^ Department of Ear-Nose-Throat, The Second People’s Hospital of Huai’An, Huai’An, P R China; ^3^ Department of Neurosurgery, Jiangning Hospital Affiliated with Nanjing Medical University, Nanjing, P R China; ^4^ Department of Neurosurgery, Jinling Hospital, School of Medicine, Nanjing University, Nanjing, P R China

**Keywords:** pituitary adenomas, bilateral nostrils, sphenoid sinus, neuroendoscopy

## Abstract

Here we review the technical aspects of our experience with the neuroendoscopic bilateral nostril (binostril) transsphenoidal approach for pituitary adenomas. A total of 42 patients were treated in our hospital from September 2013 to December 2015. Total tumor resection was completed in 31 cases, nearly full resection was achieved in 9 cases, and partial resection was achieved in 2 cases. In most cases clinical symptoms were relieved after surgery. These included 18/22 cases with visual field and vision disorders; 19/25 cases with headaches; 11/15 cases where high baseline PRL returned to normal levels; 6/7 cases where elevated blood GH returned to normal levels; and 2/3 cases where elevated blood ACTH returned to normal levels after surgery. Postoperative complications were observed in 13 patients: 8 cases of diabetes insipidus, 4 cases of cerebrospinal fluid rhinorrhea, and 1 case of subarachnoid hemorrhage. Among the key advantages of the neuroendoscopic binostril transsphenoidal approach for pituitary adenoma resection are its minimally-invasive nature, clear exposure of the operative field, high full-excision rates, improved peri-operative safety, and minor patient trauma with fewer postoperative complications.

## INTRODUCTION

Pituitary adenomas are common benign tumors of the pituitary gland, accounting for 10% of all intracranial tumors. Their incidence in unselective autopsies is as high as 20-30% [1]. In the past, the endoscopic transsphenoidal approach or transfrontal basal approach was mainly adopted for its excision [2]. Although several studies explored the pathogenesis of pituitary adenomas in order to seek new treatments [3,4], no new molecular targeted therapies were identified. In recent years, with the rapid development of the endoscopic technique, endoscopic neurosurgery has become one of the most active fields of neurosurgery, with the transsphenoidal approach through the bilateral nostrils for resection of pituitary adenomas representing perhaps the most mature and widest application of this surgical technique [5]. From September 2013 to December 2014, our hospital conducted 42 cases of pituitary adenoma resection using transsphenoidal endoscopy through the bilateral nostrils and achieved good effect. In this report, we perform an in-depth evaluation of this technique and highlight the operating characteristics and clinical outcomes.

## MATERIALS AND METHODS

### General information

Endonasal transsphenoidal endoscopy to remove pituitary adenomas was performed in 42 patients from September 2013 to December 2015 in our hospital. Patients included 18 men and 24 women, aged between 27 and 73 years, with an average age of 47.6 years. Among the 42 cases, 7 (16.7%) had growth hormone (GH) cell adenomas; 15 had prolactin (PRL) cell tumors (35.7%); 3 (7.1%) had adrenocorticotropic hormone (ACTH) cell tumors; 4 (9.5%) had mixed type tumors; and 13 (31.0%) had nonsecretory adenomas. There were 4 cases of microadenoma (diameter <1 cm), 33 cases of macroadenoma (diameter >1 cm), and 5 cases of giant adenoma (diameter >3 cm). Major clinical manifestations included impaired vision, headaches, and endocrine abnormalities.

### Surgical methods

**Figure 1:**
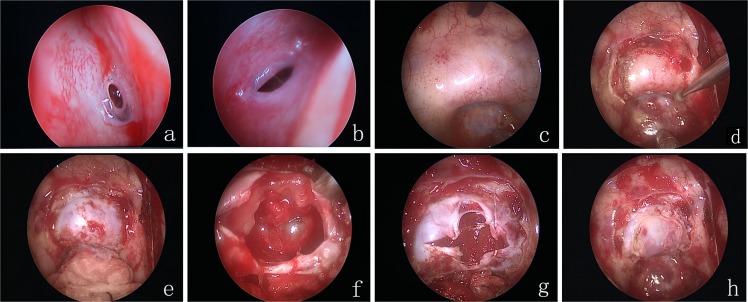
(**a**) right sphenoid sinus opening; (**b**) left sphenoid sinus opening; (**c**) opening of the sphenoid anterior wall; (**d**) removal of the sellar bone; (**e**) exposing the sellar dura; (**f**) resecting the tumor; (**g**) suturing the sellar dura by barbed wire; (**h**) resetting the sellar bone.

After successful general anesthesia, the patient was kept in the supine position, with the upper torso elevated 30°, the head turned 30° toward the left shoulder, head leaning back slightly. After the face disinfection with iodine, the patient’s bilateral sphenoid sinuses were revealed, the nasal cavity was disinfected, and a 0° endoscope (4 mm in diameter) was introduced using the bilateral nostrils as entry points. First, 0.1% adrenaline muslin as packed into the recessus sphenoethmoidalis along the nasal passages through the right nostril under endoscopic guidance, pressing it for 3∼5 minutes; the middle and inferior turbinates were exposed; the sphenoid sinus opening was found in the recessus sphenoethmoidalis between the middle turbinate and nasal septum; the sphenoid sinus opening could also be found by navigating approximately 1.5 cm above the upward side of the nostrils’ back edge. Using a unipolar electrotome, an arc incision was conducted on the nasal septum from the sphenoid sinus opening to the choana mucosa and downward toward the inferior turbinate, so as to fully reveal the inferior wall bone substance in front of the sphenoid sinus. This bone substance was grinded with a micro drill, along with the rear of the nasal septum and the sphenoid sinus septum from the sphenoid sinus openings, so as to fully expose the sphenoid sinus and shave its mucosa. A piece of bone could be kept for reconstruction of the sella bottom. The bony prominence of the sphenoid sinus cavity was revealed; after visual confirmation of the sella bottom, the bone substance at the bottom of the sella was grinded to about 1.5 cm in diameter with a high-speed drill, and both sides were exposed to the cavernous sinus; an excision was conducted on the tuberculum sellae to expose the intercavernous sinuses; below clivus exposed the intercavernous sinuses beneath. Fine needle puncture was conducted into the dura mater at the bottom of the sella. After suctioning and confirming it was safe to proceed, fulguration was carried out and a cross-shaped incision was performed at the meninges underlining the sella. During the operation, one assistant gripped the endoscope; the surgeon hold the suction tube with one hand and used a scraping ring or pituitary adenoma forceps for the curettage or excision of the tumor. Then, the surgeon positioned the endoscope at 30° into the sella to observe and excise any residual tumor. Diaphragma sellae subsidence was visible after tumor resection. A gelatin sponge was used for hemostasis of the tumor cavity by compression. During the operation, in patients who had complete diaphragma sellae and no cerebrospinal fluid leakage, a gelatin sponge was used for packing the tumor cavity; for patients who had damaged diaphragma sellae and cerebrospinal fluid leakage, fat was taken for packing and then a gelatin sponge was used for compression; finally, biological glue was used to seal it. If there was an osteocomma available, it could be implanted into the dura mater so as to support stuffing and reconstruction of the sella bottom. Expansion sponges were packed into the nasal cavity and taken out after three days. Intraoperative images are shown in Figure 1.

## RESULTS

### Surgical results

**Figure 2:**
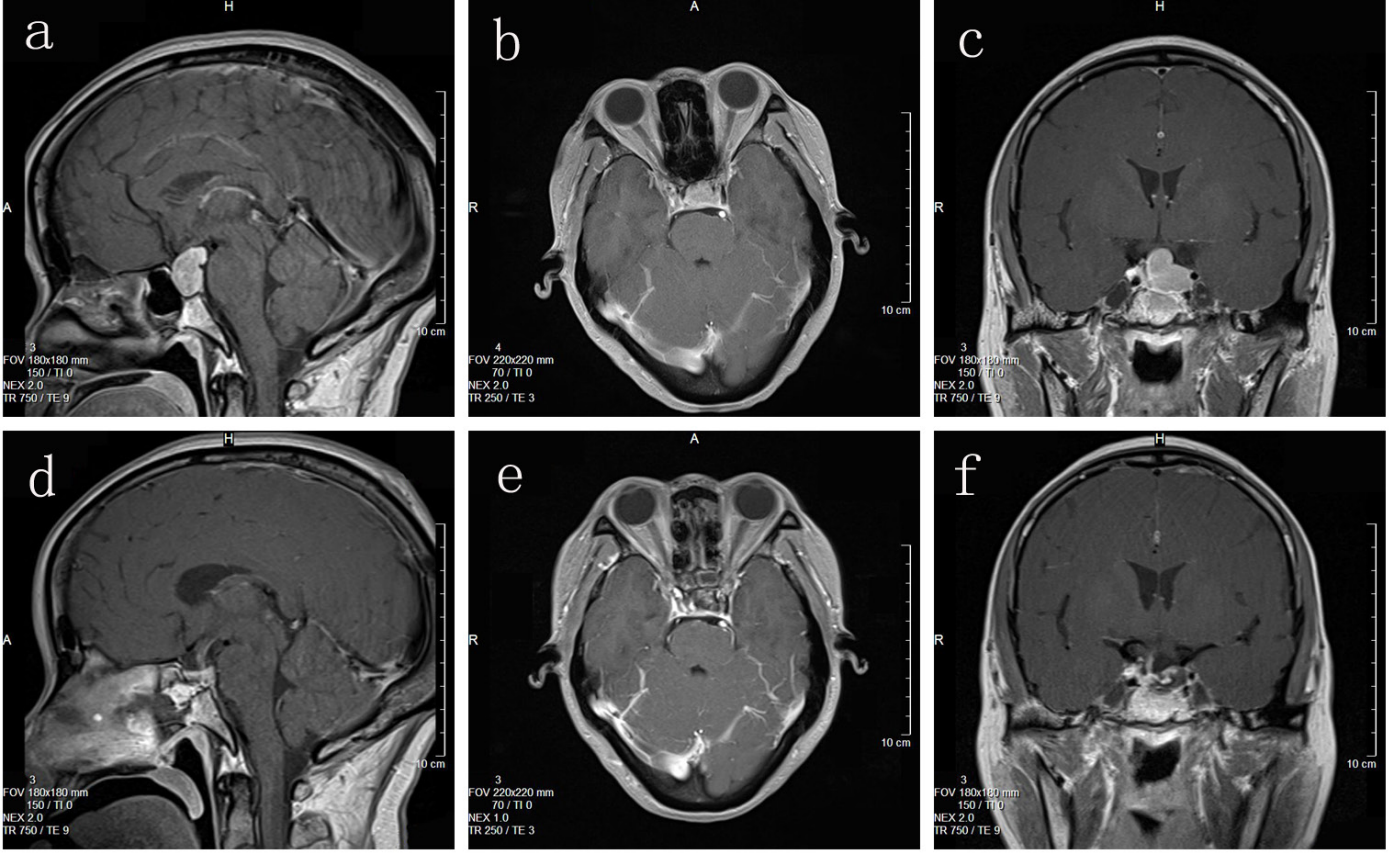
(**a, b, c**) Preoperative MRI imaging; (**d, e, f**) Postoperative MRI imaging

31 cases had total tumor excision; 9 cases had subtotal resection; 2 cases had partial resection. Postoperative symptoms improved to varying degrees: 18 of 22 patients with vision disorders and 19 of 25 patients with headaches improved significantly; of 15 patients with increased preoperative PRL, 11 regained normal levels; of 7 patients with elevated blood GH, 6 attained normal levels; of 3 patients with increased blood ACTH levels, normal levels were reestablished in 2; among the 4 mixed-type tumor patients, 3 regained normal hormone levels. Preoperative and postoperative MRI images are shown in Figure 2.

### Postoperative complications

There were 8 cases of temporary excessive urination and the symptom was improved by rehydration, application of pituitrin via intramuscular injection, or by administering desmopressin (Minirin). There were 4 cases of cerebrospinal fluid leakage resolved after 1-2 weeks of continuous drainage by a lumbar catheter; there was 1 case of subarachnoid hemorrhage, discharged after a 2-week rehabilitation treatment.

## DISCUSSION

Although pituitary adenomas are benign, they have a high recurrence rate, which is generally believed to result from incomplete resection. Cappabianca et al. estimated that surgical treatment of pituitary tumors had a 30% recurrence rate, while incomplete tumor resection was associated a recurrence rate as high as 75% [6]. Thus, for patients with pituitary adenomas complete resection of the tumor is critical. However, complete resection via the endonasal transsphenoidal endoscopic approach is sometimes difficult, as tumors are often invasive, growing within or beyond the sella, involving the dura mater or cavernous sinuses, and/or have a tough texture and may present irregular shapes. According to recent literature, the endoscopic approach for pituitary adenomas -including prolactinomas- can achieve a complete resection rate of 77%-96% [7]. Although this neurosurgery technique has many advantages, including little patient trauma, a wide operative view, a low recurrent bleeding risk, short average hospitalization time, fast recovery, etc. [7], the authors believe that the core advantages lie in its lighting features, wide endoscopic angle and fisheye effects calculations, which provide a good working angle of view that allows acquiring panoramic images. This is convenient to observe the sphenoid sinus clearly, to identify the relevant anatomical structures, and upon close tumor exploration, excise the tumor as much as possible to reduce tumor recurrence.

Here, we summarize our experience with endonasal endoscopic transsphenoidal surgery using a bilateral nostrils approach in 42 pituitary adenoma cases. Postoperative MRI review showed good overall therapeutic effect, with total tumor excision achieved in 31 cases. One of the key points of the surgery is locating the sphenoid sinus openings accurately. Each ostium is located in the posterosuperior part of sphenoid sinus recess between the inferior margin of the middle turbinate and nasal septum, but this position varies sometimes. Therefore, when it is difficult to locate, surgeons can look for it upwards the nostrils. In our patients, the lower edge of the sphenoid ostium was 15.4 ± 1.8 mm from the choanal edge; the vomer can serve as a midline landmark to locate the sphenoid sinus. Surgeons can open the sphenoid sinus cavity, remove the sphenoidal septum and identify the related anatomic structures. The superior wall is the sphenoid platform; the back wall is sella nodules recess and the sella bottom; the inferior wall is clivus; on both sides are the internal carotid artery bulge, the optic nerve bulge, and the internal opticocarotid recess. The anterior wall of the sphenoid sinus should be fully excised so as to widely expose the dura mater of the bottom of the sella. Besides, the sella bottom should be clearly identified so as to avoid the anterior skull base and clivus from being mistaken as the sella bottom. Neuronavigation or intraoperative C arm machine could assist in positioning.

During tumor resection, the tumor in the sella should be first excised, followed by its two sides, then the rear upper part and finally the front upper part so as to make sella internal decline evenly and avoid the early decline of diaphragma sellae from causing residual tumor. With the aid of preoperative MRI image analysis, the tumor’s hardness degree was preliminarily judged and intraoperative contingencies were anticipated. Pituitary tumor tissue was amorphous or fish-shaped; during tumor resection, the operator held the aspirator, curet, or microscopic tumor forceps to perform tumor resection en bloc. Some tumors have firm texture, and adhere tightly to the dura mater, so powerful ripping and dulling should be avoided. During the operation, attention should be paid to protect the normal pituitary gland and the pituitary stalk, to minimize the occurrence of postoperative refractory diabetes insipidus. In addition, a 30° endoscope could be used to stretch the tumor cavity for tumor excision completely, and any residual tumor should be excised as completely as possible.

Intraoperative internal carotid artery and/or optic nerve injuries should be absolutely avoided. Intraoperative Doppler allows for easy identification of the internal carotid artery in real-time. In 2012, Litvack et al. conducted preoperative injection of indocyanine green (12.5-25 mg) in 16 patients with benign pituitary lesions; except for one case who had cross allergy to the dye and 3 technical failures of intraoperative indocyanine green endoscopy, the internal carotid artery, cavernous sinus, optic nerve and normal pituitary could be clearly observed in 12 patients during the operation [8]. Thus, vascular and nerve damage could be minimized, and complete excision rates could be increased. Recently, Hide et al. used endoscopic endonasal transsphenoidal surgery for the resection of pituitary adenoma, tuberculum sella meningioma, craniopharyngioma, chordoma, Rathke’S cyst, and dermoid cyst surgery; they adopted the indocyanine green intravital fluorescence technique, and clearly distinguished the internal carotid artery, cavernous sinus, and optic nerve, which increased the procedure’s safety and effectiveness [9].

Due to a large extent to the operative flexibility required by the unique anatomical aspects of each case, the endoscopic neurosurgery technique for pituitary adenoma is a demanding procedure that requires, through rigorous training, a high degree of coordination between surgeons and assistants. Dallapiazza et al. proposed that surgeons could achieve operative proficiency after about 60 surgeries, when operation times, intraoperative bleeding, and postoperative complications would tend to remain basically stable [10]. Our approach required three hands of two people, and sometimes even four hands were necessary to operate; this significantly increased the operation space, and the endoscope and instruments were more harmoniously utilized, resulting in a smoother procedure. Especially after entering the sella, one assistant needed to hold the endoscope while the performer held the aspirator and curet, or tumor pincers, with both hands; this cooperation allowed to easily observe the surrounding structures and to excise the tumors smoothly.

The binostril neuroendoscopic transsphenoidal approach for pituitary adenoma resection has obvious advantages; its core purposes are to reduce surgical trauma, expand tumor exposure, increase the chances of complete tumor excision under erthyphoria, and reduce tumor recurrence. With constantly improved technology and equipment, this technique will surely undergo further development and implementation.
